# Heat Stress Impairs Endometrial Function During Implantation by Regulating Autophagy in Hainan Black Goat

**DOI:** 10.3390/ani14223213

**Published:** 2024-11-08

**Authors:** Xiaoping Li, Yanyu Sun, Yi Min, Xinyu Wang, Diqi Yang, Hui Peng

**Affiliations:** School of Tropical Agriculture and Forestry, Hainan University, Haikou 570228, China; lixiaoping970@163.com (X.L.); 23220952000010@hainanu.edu.cn (Y.S.); minyi2333@163.com (Y.M.); 20213007854@hainan.cn (X.W.)

**Keywords:** goat, heat stress, embryo implantation, autophagy, tight junction

## Abstract

Heat stress (HS) is a significant factor that adversely affects the health, welfare, and productivity of domestic animals, particularly impacting embryo implantation rates. However, the effects of HS on endometrial function during the peri-implantation period in Hainan black goats remain unclear. This study explores the influence of HS on the endometrium of these goats. We collected uterine tissue samples from both control and heat-stressed goats and performed in vitro experiments using a 2 × 2 factorial design. This design included two temperature conditions (37 °C as the control and 42 °C to simulate heat stress) and two pharmacological treatments: chloroquine (CQ), an autophagy inhibitor, and rapamycin (RAPA), an autophagy activator. Our results showed that heat stress initially suppresses autophagy activity, which is subsequently enhanced with prolonged exposure. The pharmacologic modulation of autophagy, through activation or inhibition, resulted in corresponding upregulation or downregulation of the endometrial epithelial cells’ (EECs) receptivity markers. The overexpression of ATG7 partially reversed the HS-induced downregulation of these markers. Additionally, TJP1, a tight-junction marker, was degraded under the pharmacologic and genetic activation of autophagy in HS conditions but accumulated more in the EECs pre-treated with CQ. These findings suggest that autophagy plays a protective role in maintaining endometrial function under HS conditions in Hainan black goats. This study offers valuable insights into the role of autophagy in endometrial receptivity and proposes a potential strategy to mitigate the adverse effects of HS on goat reproduction.

## Simple Summary

Heat stress (HS) negatively affects the health and productivity of domestic animals, particularly influencing embryo implantation rates. This study aims to understand the effects of heat stress on endometrial function in Hainan black goats during the peri-implantation period. We collected uterine tissue samples from both control and heat-stressed goats and conducted experiments under two temperature conditions: normal (37 °C) and heat stress (42 °C), along with two pharmacological treatments—chloroquine (CQ) and rapamycin (RAPA). Our results indicate that heat stress initially suppresses autophagy activity, which increases with prolonged exposure. Modulating autophagy through these treatments altered the expression of endometrial receptivity markers. Notably, the overexpression of a specific protein partially reversed the downregulation caused by heat stress. Additionally, a tight-junction protein was degraded under certain conditions but accumulated when cells were treated with CQ. These findings suggest that autophagy plays a protective role in maintaining endometrial function during heat stress, offering insights that may help improve reproductive outcomes in goats facing heat challenges

## 1. Introduction

Embryo implantation is a critical prerequisite for successful gestation in all mammals, contingent upon the receptivity of the endometrium. Endometrial receptivity refers to the endometrium’s ability to accept an embryo for implantation. In domestic ruminants, suboptimal endometrial receptivity is a significant contributor to implantation failure [1,2]. Various factors influence endometrial receptivity, and in livestock inhabiting tropical and subtropical regions, prolonged and extreme heat is a key determinant. As global warming progresses, rising temperatures and increasingly frequent heat waves are anticipated. The current research indicates that heat stress (HS) adversely affects physiological and reproductive performance, disrupting the ovarian phosphatidylinositol-3 kinase and steroid production signaling and altering autophagy during follicular development [3,4,5]. These disruptions impair maternal homeostasis, which negatively impacts maternal–endometrial interactions, leading to developmental abnormalities and hindering successful pregnancy establishment. The Hainan black goat, a high-quality breed native to the Hainan Province, China, is known for its flavorful meat and resistance to high temperatures, humidity, and disease [6]. In recent years, summer temperatures in southern China, including the Hainan Province, have gradually increased, with some regions experiencing prolonged periods exceeding 40 °C [7,8]. Despite its heat resistance, prolonged exposure to high temperatures and humidity still subjects the Hainan black goat to significant heat stress, adversely affecting embryo implantation and pregnancy rates. The impact of HS on the pregnancy and fetal health of ruminants such as Hainan black goats is substantial, raising widespread concern about the potential risks associated with rising temperatures.

Autophagy is a critical cellular process activated by stress signals, nutrient deprivation, or infection to restore cellular homeostasis. This bulk lysosomal degradation pathway, conserved across all eukaryotic cells, clears cytosolic components, including organelles, long-lived proteins, and protein aggregates [9]. By performing this ‘housekeeping’ function, autophagy eliminates old or damaged cellular components that could disrupt homeostasis, which is especially vital for organisms under heat stress [10]. Research by Hale et al. has demonstrated that heat stress induces the expression of autophagy proteins in the ovaries, suggesting that autophagy is a potential response mechanism to HS [5]. Additionally, a study on pig skeletal muscle revealed that 6 h of heat stress increased both autophagy and mitophagy, indicating the removal of damaged mitochondria [11]. However, it remains unclear whether autophagy plays a role in the effects of heat stress on uterine function during the embryo implantation period in goats.

In this study, we investigated the effects of heat stress on the uterine tissue structures and receptivity markers during the embryo implantation period in Hainan black goats. Furthermore, we explored whether autophagy is activated in this process and examined the potential mechanisms by which autophagy may influence uterine function under heat-stress conditions.

## 2. Materials and Methods

### 2.1. Animal Treatment

This study was conducted in accordance with the ARRIVE guidelines and the Animal Welfare Act guidelines of the Ministry of Science and Technology in China. The protocols were approved by the Hainan University Animal Care and Use Committee (HNUAUCC-2023-00031). Ten healthy female Hainan black goats (approximately 2 years old) were obtained from the Hainan Botai Agricultural Development Co., Ltd. (Haikou, Hainan, China). The goats were housed under standard conditions (12 h light/12 h dark) with ad libitum access to food and water. After a one-week acclimation period, the goats were synchronized for estrus and artificially inseminated, with this day designated as day 0 of pregnancy. The goats were then randomly divided into two groups: the control group (Con) and the heat-stress group (HS). From the first day of pregnancy, the two groups were housed in different environmental conditions.

The control group was maintained at a temperature of 25–28 °C with 45% relative humidity and a Temperature–Humidity Index (THI) range of 63.7 to 65.7. The HS group was exposed to a daytime temperature of 35–39 °C (7:00–19:00), controlled by electric heaters regulated by thermostats, with the temperature increasing approximately 1 °C per hour until the maximum temperature was reached, and a relative humidity of 65%, with a THI range of 83.1 to 85.1. During the night, the HS room was allowed to cool. THI values were calculated using the formula of Buffington [12]: THI = 0.8 × T + ((RH/100) × (T – 14.3)) + 46.4, where T represents the air temperature, and RH represents the relative humidity. On the 17th day of pregnancy, both groups of goats were sacrificed, and their uterine tissues were collected for subsequent analyses, as shown in Supplementary Figure S2.

### 2.2. Histological Analysis

For the histological analysis, uterine samples were fixed in a 4% paraformaldehyde solution for 24 h, followed by paraffin embedding and sectioning at 4 μm. The sections were stained with hematoxylin and eosin (H&E) according to standard protocols. Specifically, after deparaffinization and rehydration, the sections were incubated in a hematoxylin solution for 5 to 10 min, followed by rinsing in running tap water. They were then treated with eosin solution for 30 s to 1 min. The slides were subsequently dehydrated through graded alcohols and cleared in xylene before being mounted with coverslips. Histological observations were performed using an Olympus microscope (BX53, Olympus, Tokyo, Japan) equipped with the Panoramic Viewer system (1.15.3).

### 2.3. Transmission Electron Microscopy

Transmission electron microscopy (TEM) analysis was conducted following established methods [13]. Briefly, the endometrial tissues were fixed in glutaraldehyde for 48 h, dehydrated through a graded ethanol series, and embedded in resin. Ultrathin sections were mounted on nickel grids, stained, and visualized using a JEM-1400 electron microscope (JEOL Ltd., Akishima, Tokyo, Japan).

### 2.4. Cell Culture and Drug Treatment

Human telomerase reverse transcriptase (hTERT) was used to immortalize the goat endometrial epithelial cells (EECs), while maintaining the characteristics of primary cells [14]. The EECs were seeded in culture dishes containing DMEM/F-12 medium supplemented with 10% fetal bovine serum (FBS, AusgeneX, Loganholme, QLD, Australia). Upon reaching 70–80% confluence, the EECs were cultured in fresh DMEM/F-12 medium treated with activated carbon. Subsequently, P4 (10^−7^ M, Sigma, St. Louis, MO, USA) and E2 (10^−9^ M, Sigma, St. Louis, MO, USA) were added to the medium. After 12 h of treatment, the cells were divided into a control group (cultured at 37 °C) and a heat-stress group (cultured at 42 °C) for additional 3, 6, or 12 h, while being treated with 20 ng/mL IFN-τ (Sangon Biotech Co., Ltd., Shanghai, China). For the presence of the CQ and RAPA groups, 50 µM CQ or 100 µM RAPA was added to the EECs prior to heat-stress treatment.

### 2.5. Cell Transfection and Fluorescence Measurements

The EECs expressing GFP-LC3-RFP were maintained in our laboratory, established following the method described by Yang et al. [14]. As outlined in Section 3.4, the EECs expressing GFP-LC3-RFP were treated with P_4_ and E_2_, followed by heat stress and IFN-τ stimulation. Fluorescence was observed using a fluorescence microscope (Nikon Inc., Melville, NY, USA) and quantified using Image J 1.54d software (Rockville, MD, USA). The goat *ATG7* gene was amplified and cloned into the pCD513B vector to synthesize pCD513B-ATG7. The vector was then used for the viral packaging and transfection of the EECs as previously reported [14].

### 2.6. Immunofluorescence Staining

Slides containing uterine and EECs samples were incubated with primary antibodies at 37 °C for 2 h. The primary antibodies included anti-ATG7 (Cell Signaling Technology, Inc., Danvers, MA, USA, CST 8558, diluted 1:200), anti-MAP1LC3 (Sigma L7543, diluted 1:200, Sigma Aldrich, Co., Saint Louis, MO, USA), anti-SPP1 (Wanleibio Co., Ltd., Shenyang, China, WL02378, diluted 1:150), and anti-TFEB (ABclonal A7311, diluted 1:200, ABclonal Biotechnology Co., Ltd., Wuhan, China). Following three washes with PBS, the slides were incubated for 1 h at room temperature with Alexa-labeled secondary antibodies (ABclonal Biotechnology Co., Ltd., Wuhan, China) diluted 1:500. The nuclei were counterstained with DAPI (4,6-diamidino-2-phenylindole), and the slides were observed using a fluorescence microscope (Nikon Inc., Melville, NY, USA).

### 2.7. Western Blot Analysis

The cells and tissues were collected post-treatment, washed with ice-cold PBS, and lysed using RIPA buffer (Langeco Technology Co., Ltd., Shanghai, China). Twenty micrograms of total protein were loaded into each well of a 12% SDS-PAGE gel, and the proteins were separated by electrophoresis. The proteins were then transferred onto PVDF membranes (Millipore, Bedford, MA, USA). After blocking with Tris-buffered saline containing 0.5% Tween-100 (TBST) and 10% nonfat milk for 2 h, the samples were incubated with primary antibodies: anti-TJP1 (Bioss bs-1329R, diluted 1:1000, Bioss Biotechnology Co., Ltd., Beijing, China), anti-MAP1LC3 (Sigma L7543, diluted 1:1000), anti-ATG7 (CST 8558, diluted 1:1000), anti-SQSTM1 (CST 8025, diluted 1:1000, CST, Danvers, MA, USA), anti-HOXA10 (BBI, D163629, Shanghai, China, diluted 1:300), anti-HOXA11 (ABclonal A2976, diluted 1:1000, ABclonal Biotechnology Co., Ltd., Wuhan, China), anti-ITGB1 (ABclonal A2217, diluted 1:1000, ABclonal Biotechnology Co., Ltd., Wuhan, China), anti-HSP70 (ABclonal A12948, diluted 1:1000, ABclonal Biotechnology Co., Ltd., Wuhan, China), and anti-HSP90 (ABclonal A13501, diluted 1:1000, ABclonal Biotechnology Co., Ltd., Wuhan, China). The membranes were incubated with HRP-labeled secondary antibodies at room temperature for 1 h. The protein bands were visualized using Image-Pro Plus 6.0 software (Media Cybernetics, Inc., Silver Spring, MD, USA) and quantified using Quantity One 4.0 software (Bio-Rad Laboratories, Hercules, CA, USA).

### 2.8. Statistical Analysis

Unless otherwise specified, all data are presented as the mean ± SEM. Statistical analysis was performed using SPSS version 22 (IBM-SPSS Inc., Chicago, IL, USA). Variables were analyzed using one-way ANOVA for certain groups, while a two-way ANOVA with least-significant difference (LSD) post hoc comparisons was used for groups involving heat stress and additional treatments (CQ treatment, RAPA treatment, and ATG7 overexpression). Statistical significance was defined as *p* < 0.05.

## 3. Results

### 3.1. Heat Stress Induces Structural Damage and Autophagy Activation in Goat Endometrium During Embryo Implantation

**Figure 1:**
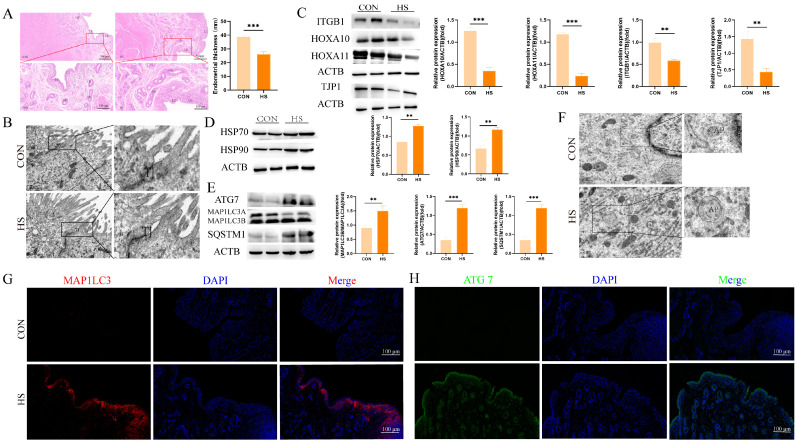
Heat stress induces structural damage and autophagy activation in goat endometrium during embryo implantation. (**A**) Hematoxylin and eosin (HE) staining of endometria from Hainan black goats on day 17 of pregnancy. (**B**) Ultrastructural images of endometria from Hainan black goats on day 17 of pregnancy (magnification: 2000×). (**C**) Western blot analysis of endometrial receptivity and tight-junction protein expression. (**D**) Western blot analysis of heat-shock protein expression. (**E**) Western blot analysis of autophagy protein expression. (**F**) Ultrastructural images showing autophagosomes and autolysosomes in the endometria of Hainan black goats on day 17 of pregnancy. (**G**) Representative merged fluorescence images of MAP1LC3 in the endometria of Hainan black goats (scale bar = 100 μm). (**H**) Representative merged fluorescence images of ATG7 in the endometria of Hainan black goats (scale bar = 100 μm). CON: control. HS: heat stress. LE: luminal epithelium. GE: glandular epithelium. TJ: tight junction. AP: autophagosome. The data are presented as the means ± SEM (** *p* < 0.01, *** *p* < 0.001).

To investigate the effects of heat stress (HS) on the goat endometrium during embryo implantation, we performed hematoxylin/eosin (H&E) staining and analyzed the epithelial thickness. The H&E staining results revealed that the luminal epithelia in heat-stress (HS)-exposed goat uteri were injured, incomplete, and exhibited shedding, in contrast to the neatly arranged and undamaged epithelial cells in the control group (Figure 1A, *p* < 0.001). To further investigate the impact of heat stress on the endometrial structure, transmission electron microscopy was employed. As shown in Figure 1B, tight junctions in the endometrial epithelial cells were expanded in the HS group, while they remained tight and intact in the control group.

Subsequently, Western blot analysis showed a decrease in the expression levels of the endometrial receptivity markers ITGB1 (*p* < 0.01), HOXA10 (*p* < 0.001), and HOXA11 (*p* < 0.001) in the HS group as compared to the controls (Figure 1C). Additionally, the tight-junction marker TJP1 was expressed at higher levels in the endometria of the control group (Figure 1C, *p* < 0.01). Heat stress also led to the increased expression of the heat-shock proteins HSP70 and HSP90 in the endometria of HS-exposed goats (Figure 1D, *p* < 0.01).

To investigate whether the autophagy was altered in the endometrium during the implantation period under heat stress, we assessed the autophagy markers MAP1LC3, SQSTM1, and ATG7. As shown in Figure 1E, enhanced expressions of MAP1LC3 (*p* < 0.01), SQSTM1 (*p* < 0.001), and ATG7 (*p* < 0.001) were observed in the HS group (Figure 1E, *p* < 0.01). Furthermore, a greater number of autophagosomes and autolysosomes were detected in the HS group (Figure 1F). Immunofluorescence staining was performed to visualize the fluorescent intensity and localization of MAPLC3 and ATG7. The results demonstrated a substantial increase in MAPLC3-positive signals in the luminal epithelia and lamina propria (stroma endometrialis) of the HS group, whereas minimal expression was noted in the control group (Figure 1G). Similarly, ATG7-positive cells were prominently observed in the luminal epithelia and glandular epithelia of the HS group (Figure 1H). These findings suggest that heat stress induces structural damage and activates autophagy in the goat endometrium during the embryo implantation period.

### 3.2. Effects of Heat Stress on Endometrial Epithelial Cell Function in Goats

**Figure 2:**
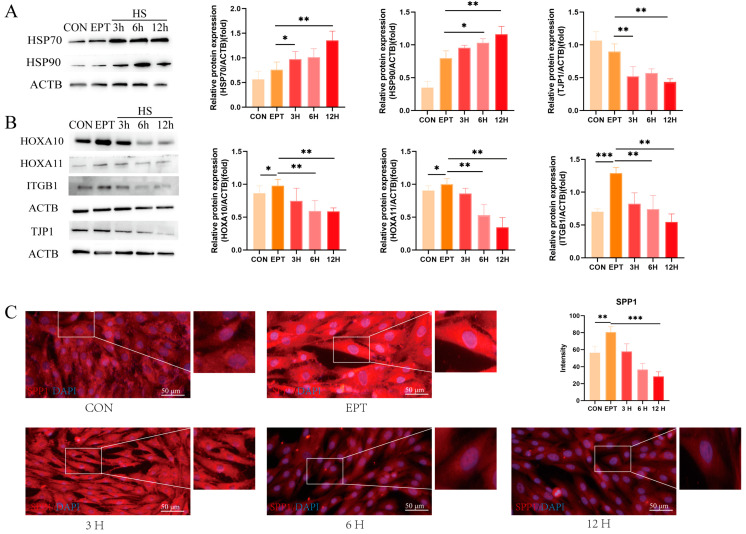
Effects of heat stress on endometrial epithelial cell function in goats: (**A**) Western blot analysis of heat-shock protein expression. (**B**) Western blot analysis of endometrial receptivity and tight-junction protein expression. (**C**) Representative merged fluorescence images of SPP1 in the EECs (scale bar = 50 μm). CON: control. EPT: progesterone, estradiol, and interferon-tau (EPT) treatment. 3 H: EECs following heat-stress treatment for 3 h with EPT pre-treatment. 6 H: EECs following heat-stress treatment for 6 h with EPT pre-treatment. 12 H: EECs following heat-stress treatment for 12 h with EPT pre-treatment. The data are presented as the means ± SEM (* *p* < 0.05, ** *p* < 0.01, *** *p* < 0.001).

To verify the impact of heat stress on the function of goat endometrial epithelial cells (EECs) during embryo implantation, we simulated the peri-implantation endometrial environment using progesterone, estradiol, and interferon-tau (EPT) and assessed the expression of endometrial receptivity markers. Initially, we examined the expression of heat-stress marker molecules over different durations of heat-stress treatment. As shown in Figure 2A, the expression levels of HSP70 and HSP90 remained consistently high with prolonged heat-stress treatment as compared to the EPT group (*p* < 0.01). Notably, as compared to the control (CON) group, the expression levels of the endometrial receptivity markers HOXA10 (*p* < 0.05), HOXA11 (*p* < 0.05), and ITGB1 (*p* < 0.001) were enhanced in the EPT group, with CON serving primarily as a comparison for the EPT group. Next, we assessed the expression of these receptivity markers, which exhibited a time-dependent decrease under heat-stress conditions (Figure 2B, *p* < 0.01), and the TJP1 expression also declined (Figure 2B, *p* < 0.01). Furthermore, we observed the fluorescence intensity of SPP1, a key cell adhesion molecule, through immunofluorescence staining. As illustrated in Figure 2C, heat stress reduced the fluorescence intensity of SPP1 in the EECs (*p* < 0.01). These results indicate that heat stress inhibits the expression of receptivity markers in endometrial epithelial cells.

### 3.3. Effects of Heat Stress on Endometrial Epithelial Cell Autophagy

**Figure 3:**
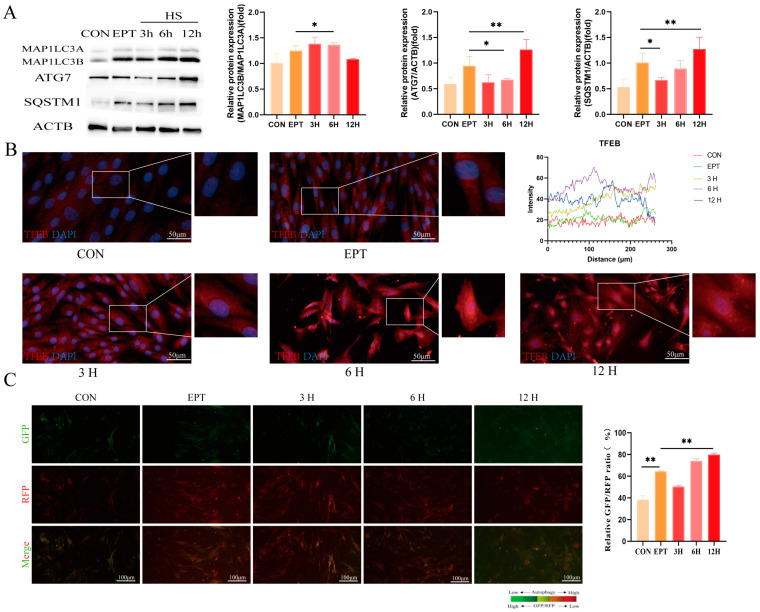
Effects of heat stress on endometrial epithelial cell autophagy: (**A**) Western blot analysis of autophagy protein expression. (**B**) Immunofluorescence staining showing the expression and localization of TFEB in the EECs under heat stress (scale bar = 50 μm). (**C**) The GFP/RFP fluorescence ratio images of the EECs following heat-stress treatment with progesterone, estradiol, and interferon-tau (EPT) pre-treatment are shown (magnification: 200×). CON: control. EPT: progesterone, estradiol, and interferon-tau (EPT) treatment. 3 H: EECs following heat-stress treatment for 3 h with EPT pre-treatment. 6H: EECs following heat-stress treatment for 6 h with EPT pre-treatment. 12H: EECs following heat-stress treatment for 12 h with EPT pre-treatment. The data are presented as the means ± SEM (* *p* < 0.05, ** *p* < 0.01).

To explore the impact of heat stress on the autophagic flux in the endometrial epithelial cells (EECs), we first examined the expression levels of autophagy markers MAP1LC3B (*p* < 0.05), ATG7 (*p* < 0.01), and SQSTM1 (*p* < 0.01). As shown in Figure 3A, the expression levels of these markers were lower in the heat-stress group after 3 h as compared to the EPT group. However, with prolonged heat-stress treatment, the expression of the autophagy markers began to increase at 6 h and was higher at 12 h than in the EPT group. The immunofluorescence results demonstrated that TFEB primarily accumulated in the cytoplasm after 3 h of heat stress but translocated to the nucleus after 6 and 12 h of treatment (Figure 3B). To further confirm these findings, we utilized the autophagy probe GFP-MAP1LC3B-RFP, which monitors autophagic flux by calculating the GFP/RFP ratio. As illustrated in Figure 3C, the GFP/RFP ratio decreased after 3 h of heat stress but increased after 6 and 12 h (*p* < 0.01). However, following 12 h of heat-stress treatment, the EECs exhibited shrinkage and necrosis (Supplementary Figure S1). Therefore, subsequent experiments were conducted using a 6-h heat-stress treatment. These results suggest that heat stress initially suppresses autophagic flux, but with prolonged exposure, autophagic activity is gradually activated, potentially as a protective cellular mechanism.

### 3.4. Effects of Modulating Autophagy Activity on Heat-Stress-Induced Damage in Goat Endometrial Epithelial Cells

**Figure 4:**
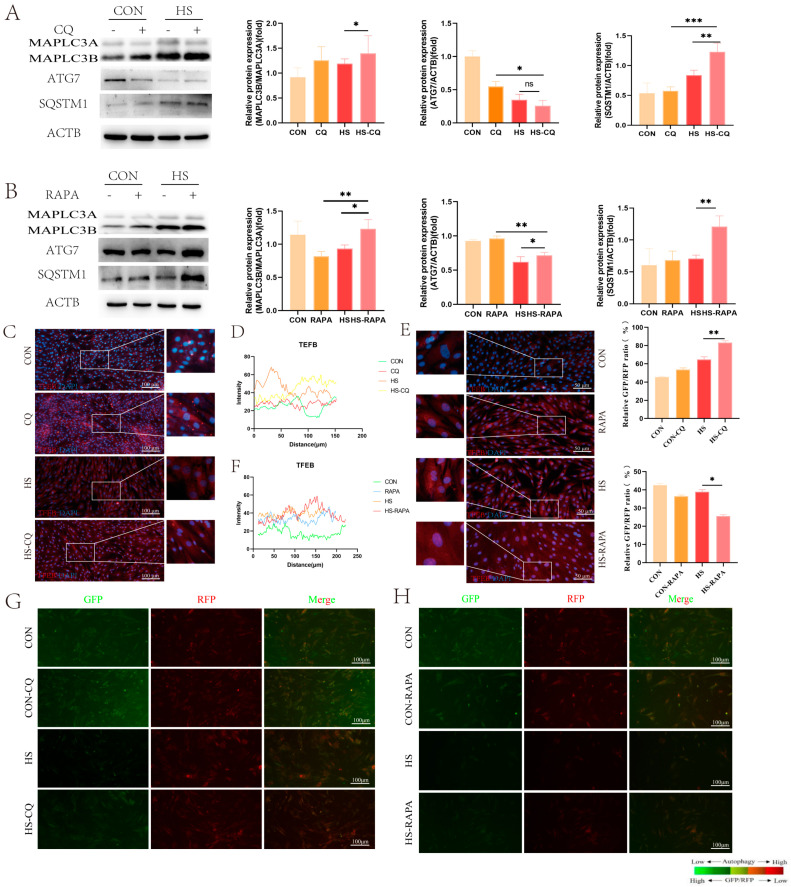
Effects of CQ and RAPA pre-treatment on autophagy protein expression in the EECs under heat stress for 6 h: (**A**) Expression of autophagy proteins in the EECs pre-treated with CQ under heat stress for 6 h. (**B**) Expression of autophagy proteins in the EECs pre-treated with RAPA under heat stress for 6 h. (**C**–**F**) Immunofluorescence staining showing the expression and localization and histograms of the fluoresce of TFEB in the EECs under heat stress with CQ or RAPA (scale bar = 50 μm). (**G**,**H**) The GFP/RFP fluorescence ratio images of the EECs following heat-stress treatment with progesterone, estradiol, and interferon-tau (EPT) pre-treatment in the presence of CQ or RAPA (magnification: 200×). CQ: Chloroquine. RAPA: Rapamycin. HS-CQ: EECs following heat-stress treatment for 6 h with CQ pre-treatment. HS-RAPA: EECs following heat-stress treatment for 6 h with RAPA pre-treatment. The data are presented as the means ± SEM (* *p* < 0.05, ** *p* < 0.01, *** *p* < 0.001, ns: not significant).

Based on the results above, we hypothesized that changes in autophagy activity induced by heat stress may regulate the function of the EECs during embryo implantation. To investigate this, we altered the autophagy activity in the EECs, using rapamycin (RAPA) to activate and chloroquine (CQ) to inhibit autophagy, respectively. Our findings indicated that both CQ and RAPA treatments enhanced MAP1LC3B levels (Figure 4A,B, *p* < 0.05), which is consistent with previous studies [15]. Additionally, the expression of SQSTM1 was higher in both the CQ and RAPA treatment groups under heat-stress conditions as compared to the control group (Figure 4A,B, *p* < 0.01). Interestingly, CQ pre-treatment did not affect ATG7 expression under heat stress, whereas Rapa increased the ATG7 levels (Figure 4A,B, *p* < 0.05). Immunofluorescence staining showed the increased nuclear fluorescence intensity of TFEB in both the CQ and Rapa treated groups (Figure 4C–F). Furthermore, the CQ pre-treatment increased the GFP/RFP ratio, while RAPA decreased the GFP/RFP ratio as compared to the heat-stress group (Figure 4G,H, *p* < 0.05).

**Figure 5:**
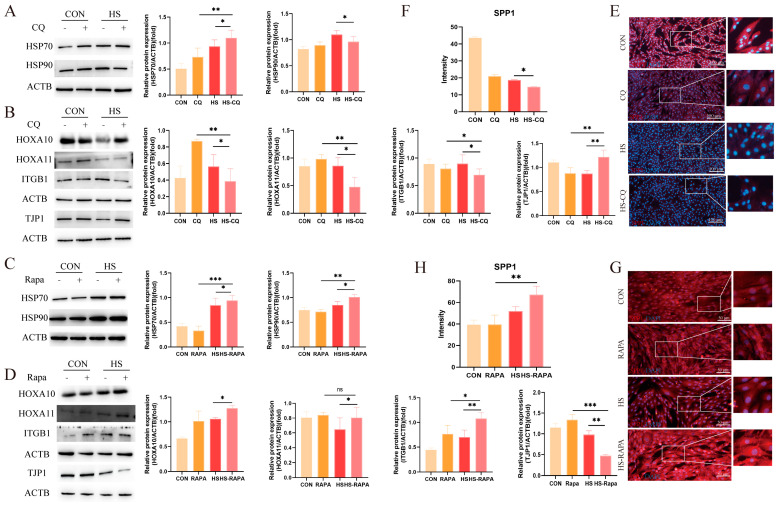
Effects of CQ and RAPA pre-treatment on receptivity markers and tight-junction proteins in the EECs under heat stress for 6 h: (**A**) Expression of heat-shock proteins in the EECs pre-treated with CQ under heat stress for 6 h. (**B**) Effects of CQ pre-treatment on the expression of receptivity markers and tight-junction proteins in the EECs under heat stress for 6 h. (**C**) Expression of heat-shock proteins in the EECs pre-treated with RAPA under heat stress for 6 h. (**D**) Effects of RAPA pre-treatment on the expression of receptivity markers and tight-junction proteins in the EECs under heat stress for 6 h. (**E**–**H**) Representative merged fluorescence images of SPP1 in EECs in the presence of CQ or RAPA (scale bar = 50 μm). CQ: Chloroquine. RAPA: Rapamycin. HS-CQ: EECs following heat-stress treatment for 6 h with CQ pre-treatment. HS-RAPA: EECs following heat-stress treatment for 6 h with RAPA pre-treatment. The data are presented as the means ± SEM (* *p* < 0.05, ** *p* < 0.01, *** *p* < 0.001, ns: not significant).

We then examined the expression of heat-stress markers HSP70 and HSP90 in the EECs pre-treated with CQ or RAPA under heat stress. Western blot analysis showed that the expressions of HSP70 and HSP90 were not altered by changes in autophagic flux (Figure 5A,C, *p* < 0.05). Under CQ treatment, the expressions of HOXA10, HOXA11, and ITGB1 were further suppressed under heat stress (*p* < 0.05), whereas the TJP1 expression was elevated (Figure 5B, *p* < 0.05). The fluorescence intensity of SPP1 decreased as compared to the control group (Figure 5E,F, *p* < 0.05). Conversely, the RAPA pre-treatment increased the expressions of HOXA10, HOXA11, and ITGB1 under heat stress, while the TJP1 expression was reduced (Figure 5D, *p* < 0.05). Additionally, SPP1 exhibited stronger fluorescence signals as compared to the control group (Figure 5G,H, *p* < 0.01). These results suggest that modulating autophagy activity affects the functional integrity of the EECs under heat stress during embryo implantation.

### 3.5. Overexpression of ATG7 Partially Restores Heat-Stress-Induced Damage in Endometrial Epithelial Cells

**Figure 6:**
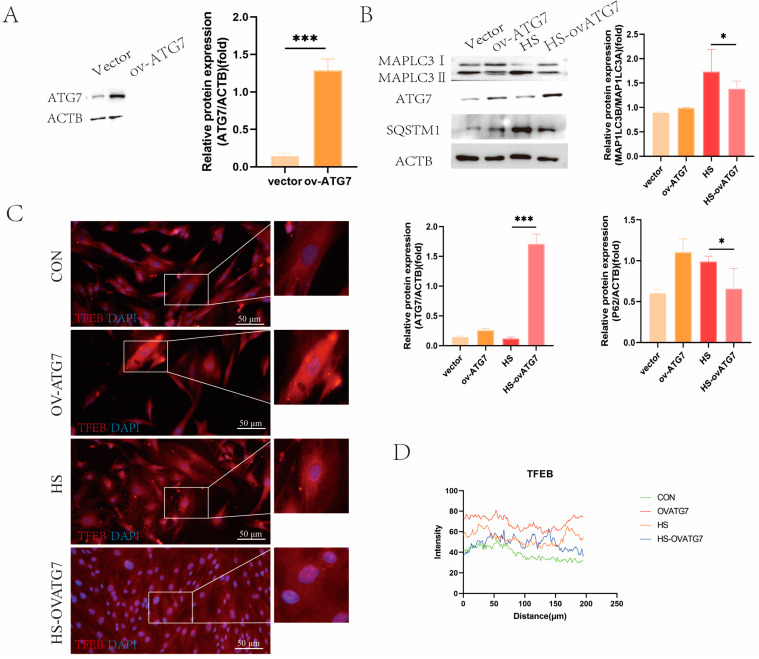
Effects of ATG7 overexpression on the expression of autophagy markers in the EECs under heat stress: (**A**) Western blot analysis of ATG7 overexpression efficiency. (**B**) Expression of autophagy proteins in ATG7-overexpressing the EECs under heat stress. (**C**,**D**) Immunofluorescence staining showing the expression and localization of TFEB in ATG7-overexpressing the EECs under heat stress (scale bar = 50 μm). OV-ATG7: overexpression of ATG7. HS-OVATG7: EECs following heat-stress treatment for 6 h with OVATG7 pre-treatment. The data are presented as the means ± SEM (* *p* < 0.05, *** *p* < 0.001).

To further validate the role of autophagy in regulating the EECs’ function under heat stress, we employed the overexpression of ATG7, a key autophagy protein. As shown in Figure 6A, lentiviral infection increased the ATG7 expression in the EECs (*p* < 0.001). The overexpression of ATG7 notably reduced the MAP1LC3B/MAP1LC3A ratio and SQSTM1 expression under heat-stress conditions (Figure 6B, *p* < 0.05). Additionally, increased nuclear TFEB signals were observed in the EECs overexpressing ATG7 under heat-stress treatment (Figure 6C,D).

**Figure 7:**
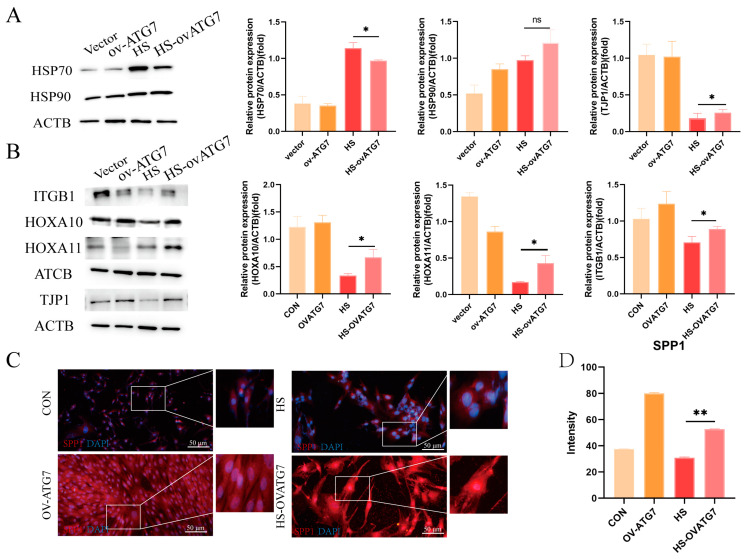
Effects of ATG7 overexpression on the expression of receptivity markers and tight-junction proteins in the EECs under heat stress. (**A**) Expression of heat-shock proteins in the ATG7-overexpressing EECs after 6 h of heat stress. (**B**) Expression of receptivity markers and tight-junction proteins in the ATG7-overexpressing EECs after 6 h of heat stress. (**C**,**D**) Representative merged fluorescence images and histograms of the fluorescence intensity of SPP1 in the ATG7-overexpressing EECs (scale bar = 50 μm). OV-ATG7: overexpression of ATG7. HS-OVATG7: EECs following heat-stress treatment for 6 h with OVATG7 pre-treatment. The data are presented as the means ± SEM (* *p* < 0.05, ** *p* < 0.01, ns: not significant).

Consistent with the effects of RAPA on heat-shock proteins, the overexpression of ATG7 did not affect the expression of HSP90, but it inhibited the expression of HSP70 (Figure 7A, *p* < 0.05). However, the overexpression of ATG7 restored the EECs’ function under heat stress, as evidenced by the upregulation of ITGB1, HOXA10, and HOXA11 protein expressions (Figure 7B, *p* < 0.05). Moreover, Western blot analysis revealed that ATG7 overexpression promoted TJP1 degradation (Figure 7B, *p* < 0.05). Immunofluorescent microscopy demonstrated that the decreased fluorescent intensity of SPP1 in the heat-stressed EECs was reversed by ATG7 overexpression (Figure 7C,D, *p* < 0.01). These results suggest that ATG7-mediated autophagy can partially restore the functional integrity of the EECs under heat stress.

## 4. Discussion

Peri-implantation events, including apposition and adhesion between the maternal endometrial surface and the elongating conceptus, are essential for successful fetal and placental development [16]. For livestock, particularly ruminants in open environments, the readiness of the uterus for implantation is influenced by both internal factors (such as age and uterine environment) and external environmental conditions. With global warming and rising temperatures, understanding how heat stress (HS) impacts reproductive systems has become increasingly important [17]. Despite this, the specific effects of HS on endometrial function during the peri-implantation period in Hainan black goats remain underexplored. Our study provides new insights into how HS affects the endometria in Hainan black goats. We observed that HS induces significant structural changes, including reduced endometrial thickness and disrupted epithelial tight junctions. These findings align with previous research that showed that HS can impair the uterine conditions critical for embryo implantation. For example, Lian et al. reported reduced luminal epithelial thickness and compromised microvilli in heat-stressed pigs [18]. Similarly, Han et al. noted structural disruptions in the endometrial epithelium of heat-stressed female rats [19]. Our results are consistent with these studies, suggesting that HS negatively impacts endometrial thickness and barrier integrity, which may hinder successful implantation.

Autophagy is a cellular process crucial for maintaining homeostasis, wherein autophagosomes sequester, recycle, and degrade proteins and cytoplasmic constituents. Under various environmental stimuli or damage, autophagy is often activated to help the organism adapt and recover [20]. Autophagy-related 7 (ATG7) functions as an E1-like activating enzyme that facilitates autophagosome formation through the ATG5–ATG12 ubiquitin-like conjugation pathways [21]. Transcription factor EB (TFEB), a member of the microphthalmia-associated transcription factor (MITF)/transcriptional factor E (TFE) family, is a master regulator of lysosomal function and autophagy, controlling lysosomal biogenesis [22]. During autophagy, MAP1LC3A is cleaved and conjugated to phosphatidylethanolamine to form MAP1LC3B, a marker of autophagosomes [23]. p62/SQSTM1 acts as a major cargo receptor for the selective degradation of misfolded ubiquitinated proteins, known as autophagy [24]. These proteins are commonly used to assess autophagy levels. In this study, the in vivo results showed that heat stress enhanced the expression of autophagy markers in the endometrium during the peri-implantation period. ATG7- and MAP1LC3-positive cells were widely observed in the endometrium, aligning with findings in other tissues. For instance, Perez–Hernandez et al. reported a significant increase in the MAP1LC3 expression in the mammary glands of heat-stressed cows [25]. In male reproductive studies, heat stress upregulated autophagic flux in testicular Sertoli cells, as evidenced by increased MAP1LC3B and LAMP1 levels and decreased SQSTM1 levels [26]. Interestingly, our in vitro experiments revealed that short-term heat stress initially suppressed autophagy, which was subsequently enhanced with prolonged heat exposure. This is consistent with findings by S. Ganesan et al., who reported that short-term heat stress (12 h) inhibited autophagy in pig skeletal muscle, as marked by decreased MAP1LC3A/MAP1LC3B levels and the activation of upstream autophagy pathways [11]. However, extending heat exposure to 24 h upregulated the autophagy markers in the right ventricular tissues of pigs [27]. We hypothesize that prolonged HS activates autophagy as a protective mechanism, helping cells cope with stress-induced damage.

The pharmacological modulation of autophagy further supports this hypothesis. We found that activating autophagy with rapamycin alleviated the negative effects of HS on the endometrial epithelial cells’ (EECs) receptivity markers, while inhibiting autophagy with chloroquine exacerbated these effects. These results suggest that autophagy is involved in the regulation of endometrial function under heat stress. To our knowledge, there are no previous studies specifically linking autophagy to endometrial function regulation during embryo implantation under heat-stress conditions. However, numerous studies have shown that autophagy plays a role in regulating endometrial receptivity during the implantation period [28,29,30]. For instance, Wang et al. reported that women with recurrent implantation failure exhibited low expressions of autophagy markers in the endometria. In vitro treatment with hCG increased the expression of receptivity markers such as FOXO1 and HOXA10, accompanied by enhanced autophagic flux, although the role of autophagy in hCG-mediated receptivity regulation was not explored [31]. Another study found that electroacupuncture treatment improved uterine receptivity in a rat model of a mechanical injury combined with lipopolysaccharide infection by increasing the levels of LC3 and Beclin1 and decreasing the levels of p62. This therapeutic effect was reversed by the autophagy inhibitor 3-methyladenine [32]. Similar to our findings with pharmacological autophagy activation, the overexpression of ATG7 partially restored the receptivity phenotype in the EECs affected by heat stress. This aligns with a study in goat endometrial epithelial cells, where P_4_, E_2_, and IFN-τ activated autophagic flux and the overexpression of ATG7 could rescue receptivity defects caused by CRIM1 deficiency [14]. Regarding the tight-junction structures in the EECs, treatment with RAPA and the overexpression of ATG7 decreased the expression of TJP1, whereas CQ upregulated TJP1. Although TJP1 is a crucial component of tight-junction structures, its deficiency may be detrimental to tight-junction integrity [33]. However, as previous studies have shown [15,34], autophagy can facilitate the recycling and degradation of damaged tight-junction proteins within the cytoplasm, which is beneficial for the formation of new tight junctions. This might explain the observed changes in the TJP1 expression in our in vitro experiments. Collectively, these findings demonstrate that heat stress impairs endometrial receptivity in Hainan black goats and that modulating autophagy may be a potential therapeutic strategy for restoring receptivity lost due to heat stress.

## 5. Conclusions

**Figure 8:**
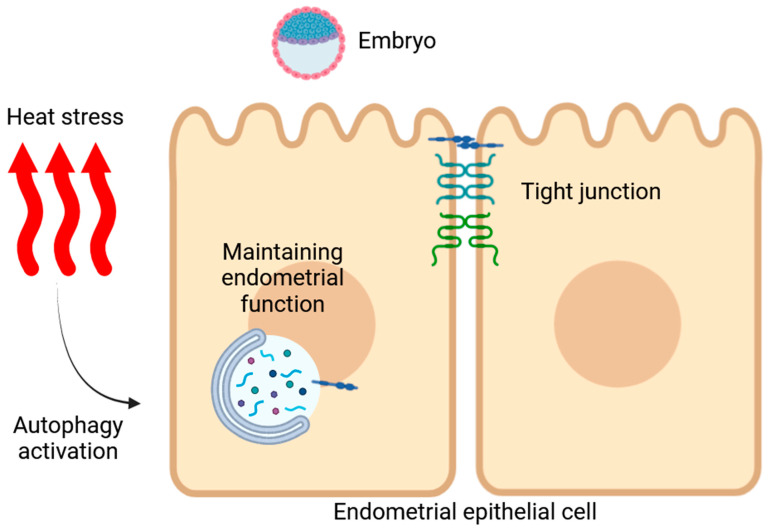
Schematic diagram illustrating heat-stress-induced damage to the endometrial function in Hainan black goats, highlighting the role of autophagy in regulating the EECs’ function.

In conclusion, this study confirms that HS impairs the structural integrity of the endometria during the peri-implantation period in Hainan black goats. Additionally, HS elevates autophagy levels in the endometrium during implantation. In vitro studies revealed that HS initially suppressed autophagy activity but enhanced it with prolonged exposure. The pharmacologic and genetic activation of autophagy partially restored the expression of receptivity markers in the EECs and facilitated the degradation of TJP1 (Figure 8). Future research should aim to elucidate the specific molecular mechanisms through which autophagy confers protection to endometrial function and to explore potential interventions that could improve reproductive success under conditions of thermal stress.

## Data Availability

The data that support the findings of this study are available from the corresponding author upon reasonable request.

## Supplementary Materials

The following supporting information can be downloaded at https://www.mdpi.com/article/10.3390/ani14223213/s1: Figure S1: Changes in cell state after heat stress. Figure S2: Overall experimental workflow. File S1: WB original images.
