# Supplementary material for: Heat Stress Impairs Endometrial Function During Implantation by Regulating Autophagy in Hainan Black Goat
Source: Animals (Basel). 2024 Nov 8;14(22):3213. doi: 10.3390/ani14223213 (PMC11591286; doi:10.3390/ani14223213)

Supplementary Figure S1: Changes in cell state after heat stress

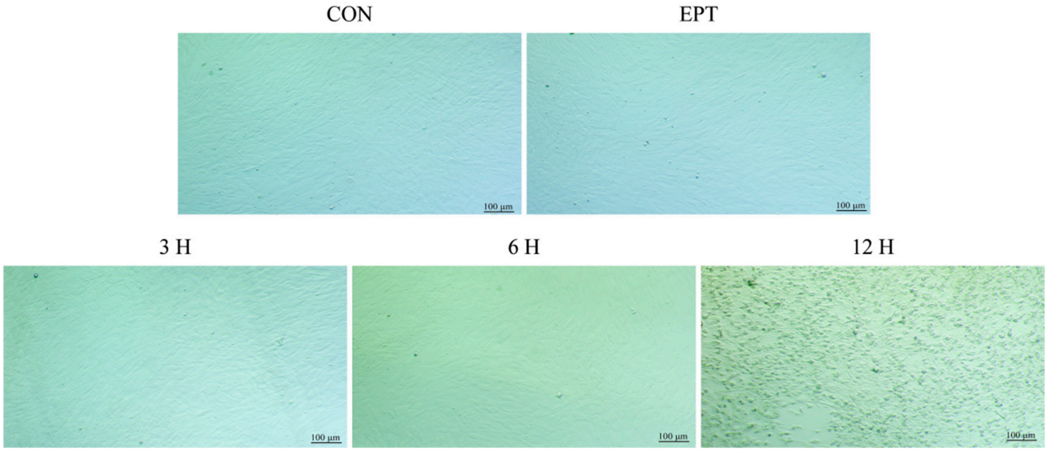

Supplementary Figure S2: Overall experimental workflow

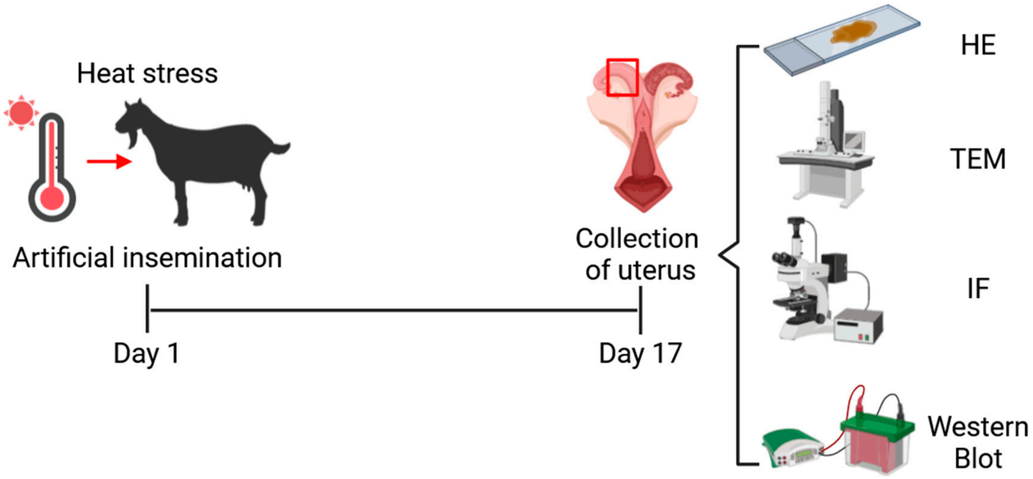

Supplement: Supplementary file 1 [file animals-14-03213-s001.zip › animals-3283739-supplementary.pdf]
